# Statistical nonParametric Mapping Enables Rigorous Comparison of Collagen Fibril Diameter Distributions

**DOI:** 10.1007/s10439-026-04064-2

**Published:** 2026-03-04

**Authors:** Jeremy D. Eekhoff, Louis J. Soslowsky

**Affiliations:** McKay Orthopaedic Research Laboratory, University of Pennsylvania, Philadelphia, PA USA

**Keywords:** Statistical nonparametric mapping, Distributional data, Collagen fibrils, Extracellular matrix, Transmission electron microscopy

## Abstract

**Purpose:**

Collagen fibrils provide mechanical integrity to the extracellular matrix in a variety of biological tissues. In biomedical research, quantification of fibril diameters is essential to describe remodeling of the matrix that can occur during development, disease, and healing. However, current statistical methods to analyze differences in the distribution of fibril diameters have significant limitations. This study evaluated a rigorous alternative method, Statistical nonParametric Mapping (SnPM), to compare fibril diameter distributions.

**Methods:**

Randomly generated simulated datasets and experimental datasets of fibril diameter distributions were analyzed using both conventional tests and SnPM. Results from each method were compared.

**Results:**

Conventional statistical tests to compare fibril diameter distributions demonstrated limitations. Comparison of average diameters detected overall shifts in distributions but not more nuanced changes, while analysis of binned relative frequencies showed dependency on the choice of bin width and location. In contrast, comparison of kernel density estimated probability density functions using SnPM both detected differences between groups and located those differences to specific ranges of fibril diameters.

**Conclusion:**

Comparative analysis of groups of fibril diameter distributions using SnPM overcomes the limitations of current techniques and provides a reliable and rigorous technique for these data. Other potential applications for SnPM in biomedical research abound, extending to other distributional or multidimensional data.

**Supplementary Information:**

The online version contains supplementary material available at https://doi.org/10.1007/s10439-026-04064-2.

## Introduction

Fibrillar collagens are key structural components of extracellular matrix, providing strength and mechanical integrity to biological tissues. During fibrillogenesis, individual collagen alpha chains come together to produce the triple helix structure of collagen molecules, which are cross-linked together in a quarter-staggered formation to form collagen fibrils [1, 2]. These fibrils function as long nanoscopic cables that deliver excellent resistance to tensile forces and provide mechanical integrity to the tissue. The specific function and properties of the extracellular matrix can vary widely between different tissues depending on the structure and organization of the collagen fibrils. Even within the same tissue, some fibril populations may play different roles than others [3]. Moreover, changes in the fibrillar matrix during development, disease, and healing have functional consequences for the tissue. Therefore, it is important to have tools to quantify and compare the structure of collagen fibrils, such as changes in fibril diameters under different experimental conditions.

The sub-micron size of collagen fibrils prohibits visualization using optical techniques, so scanning probe or electron microscopy are typically used to image and measure fibrils. In one common application, automated analysis of transmission electron micrographs is used to quantify fibril diameters from a collection of images from a single piece of tissue. This analysis generates up to thousands of collagen fibril diameter measurements for each biological sample. This abundance of data can complicate statistical comparisons between experimental groups, since conventional 0-dimensional statistical tests, such as a t-test or analysis of variance (ANOVA), compare groups with one independent scalar value per sample, rather than a full distribution of distinct observations that are not readily reduced down to a single value (e.g., mean value) without loss of meaningful information [4]. Unfortunately, this data reduction leads to statistical analysis that can fail to fully capture differences in fibril diameter distributions between experimental groups, and therefore alternative statistical techniques that could overcome this limitation should be explored.

A better approach may be to adopt a statistical test that can be used to compare groups of continuous distributions, rather than using more familiar tests that compare scalar values. Statistical parametric mapping (SPM) is a n-dimensional technique that has been used widely to compare groups of functional magnetic resonance images, and more recently, in the biomechanics field, to compare time series or other multidimensional data [5, 6]. A similar nonparametric approach, statistical nonparametric mapping (SnPM), has also been developed that uses permutation-based inference to calculate the critical test statistic, bypassing the assumptions required for parametric testing [7]. Recent examples of how SnPM is being used include comparing regional differences in brain activity during different activities measured using electroencephalography, or determining how factors such as muscle strength and fatigue impact the kinetics and kinematics of the lower limbs throughout the gait cycle using data from experiments and computational models [8–11].

The objective of this study was to determine the utility of using SnPM to compare fibril diameter distributions between experimental groups in comparison to previously used methods. We demonstrate that comparative analysis of fibril diameter distributions using SnPM produces reliable and rigorous results, overcoming the limitations of alternative techniques. While our focus in this paper is on the analysis of collagen fibril diameters, this technique has broader applicability within biomedical engineering to similar distributional data, or other types of n-dimensional data.

## Methods

### Simulated and Experimental Datasets

Simulated datasets were generated that approximated measurements of collagen fibril diameters. For each sample within the artificial dataset, 100 datapoints were randomly sampled from a unimodal or bimodal Gaussian distribution (Table 1), which was determined to be sufficient to produce a distribution of values that appropriately simulated experimental data. Variability between samples within the simulated datasets was simulated by (1) shifting the underlying distribution for each sample by a random value and (2) adding a random value to each individual observation for that sample. Eight samples were generated per group, and there were two groups generated per dataset that had different underlying distributions.

**Table 1:** Underlying probability density functions of simulated datasets

Unimodal simulated dataset	Group 1	$f\left(x\right)= \frac{1}{22.5\sqrt{2\pi }}{e}^{-\frac{1}{2}{\left(\frac{x-101.25}{22.5}\right)}^{2}}$
	Group 2	$f\left(x\right)= \frac{1}{22.5\sqrt{2\pi }}{e}^{-\frac{1}{2}{\left(\frac{x-90}{22.5}\right)}^{2}}$
Bimodal simulated dataset	Group 1	$f\left(x\right)= 0.6\frac{1}{20\sqrt{2\pi }}{e}^{-\frac{1}{2}{\left(\frac{x-103.5}{20}\right)}^{2}}+0.4\frac{1}{20\sqrt{2\pi }}{e}^{-\frac{1}{2}{\left(\frac{x-88.5}{20}\right)}^{2}}$
	Group 2	$f\left(x\right)= 0.6\frac{1}{20\sqrt{2\pi }}{e}^{-\frac{1}{2}{\left(\frac{x-78.5}{20}\right)}^{2}}+0.4\frac{1}{20\sqrt{2\pi }}{e}^{-\frac{1}{2}{\left(\frac{x-138.5}{20}\right)}^{2}}$

In addition to verifying the use of SnPM on simulated datasets, SnPM was also validated using experimental data from two previously published datasets. The first of the experimental datasets contained data on collagen fibril diameters measured in the insertion and midsubstance regions of the supraspinatus tendon in 150-day-old male mice [12]. In this dataset, there were 9–10 biological samples per group, and a total of 7173–12,732 fibril diameters measured per sample. The second experimental dataset contained data on collagen fibril diameters in mouse patellar tendon measured at postnatal days 10, 30, and 60 [13]. In this dataset, there were 6 biological samples per group, and a total of 3243–25,685 fibril diameters measured per sample.

### Analysis of Datasets

Within each dataset containing two groups, differences between groups were analyzed using two different statistical methods commonly used in prior studies. These analysis methods, although not exhaustive of all options, provide representative alternatives to SnPM for comparison. First, the average diameter for each sample was calculated and compared across groups using a two-tailed Student’s *t*-test. Second, the data were analyzed using binned analysis of relative frequencies. The relative frequency of the number of observations within bins, or equally spaced ranges of values, was determined on a sample-wise basis. Then, a two-tailed Student’s *t*-test was used to compare the relative frequencies between groups within each bin, with Bonferroni correction used to account for multiple comparisons. This analysis was performed with bin widths of 15, 30, and 45 nm, and with shifts in the bin locations corresponding to one-third and two-thirds of the bin width, to demonstrate how these parameters influence the result from this analysis.

Then, the same data were analyzed using SnPM. Continuous probability density functions were generated for each sample using kernel density estimation. The kernel bandwidth was determined by first pooling all the data within the dataset and calculating the optimal bandwidth using the normal approximation method [14], which was then applied individually to each sample in that dataset using a Gaussian kernel, so that all data would be treated equally and all fibril measurements would have an identical impact on the shape of the probability density functions. A one-dimensional two-tailed *t*-test with nonparametric inference was used to compare probability density functions between groups using spm1d (version M.0.4.52), an open-source package for statistical parametric and nonparametric mapping [15]. Briefly, this analysis calculates the test statistic, SnPM{t}, as a function of fibril diameter. Nonparametric inference is used to determine the critical test statistic (t*), which is the value where the absolute value of SnPM{t} exceeds t* in only α% of random permutations of the data. Nonparametric, rather than parametric, inference was used due to the probability density functions not being normally distributed, particularly near the tails of the probability density functions. By definition, if the maximum absolute value of SnPM{t} exceeds t*, the *p*-value is less than α and the null hypothesis that all the data were sampled from the same source can be rejected. In addition, individual p-values are determined for each continuous region where the absolute value of SnPM{t} exceeds t*, denoted as a suprathreshold cluster. The p-values for each suprathreshold cluster give the probability that random permutations of the data would generate a cluster with an integral area as large or larger than the observed cluster.

Statistical comparisons of average fibril diameters and binned relative frequencies were conducted similarly for the second experimental dataset that contained three groups, using one-way analysis of variance (ANOVA) and post hoc t-tests with Bonferroni corrections for pairwise comparisons. A one-dimensional ANOVA with nonparametric inference was used to compare kernel density estimated probability density functions between groups. This analysis is similar to the one-dimensional t-test described above, with the F-statistic used instead of the t-statistic. Post hoc one-dimensional t-tests were used to make pairwise comparisons between groups following the ANOVA, using Bonferroni corrections to account for multiple comparisons.

To evaluate the effect of bandwidth selection on the results from analysis, SnPM analysis for all datasets was repeated using the plug-in bandwidth selection method [16] and compared to the previous results that used the normal approximation bandwidth selection method.

All analysis was carried out using MATLAB with *α* = 0.05.

## Results

### SnPM Detects Differences in Two Groups of Randomly Generated Unimodal Distributions

**Fig. 1:**
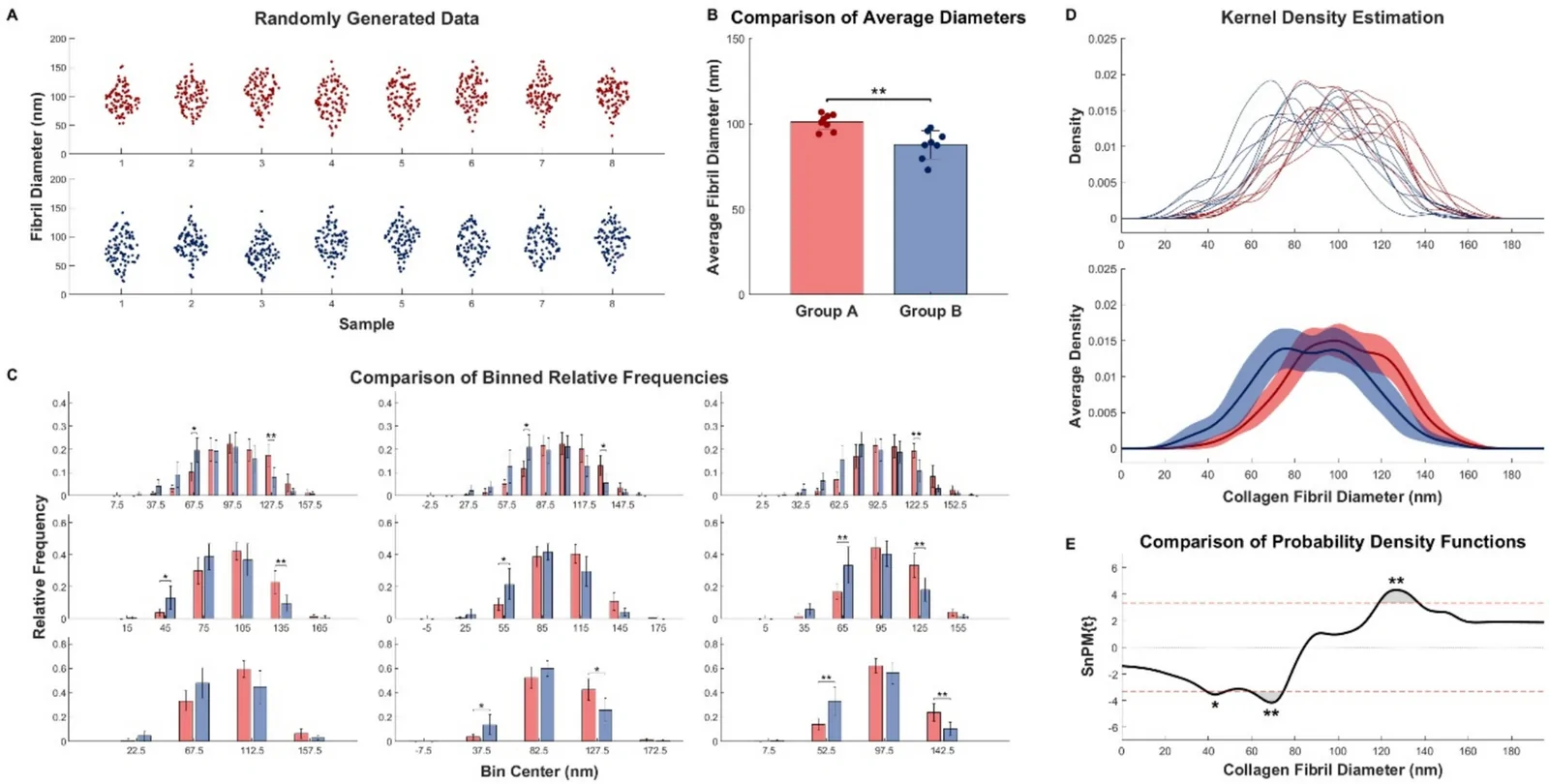
**A** Randomly generated unimodal data to simulate fibril diameter measurements. Each data point represents the diameter of a single collagen fibril, with 100 points per sample and 8 samples per group. **B** Comparison of average diameters detected a difference between groups. **C** Analysis of binned relative frequencies detected differences between groups but specific results depended on bin width and location. **D** Kernel density estimation generated sample-specific probability density functions. **E** Comparative analysis of probability density functions using SnPM detected and located differences between groups, where suprathreshold clusters are shown as continuous gray shaded areas that exceed the critical test statistic, shown as dashed red lines. Summary data shown as mean ± standard deviation. *p < 0.05, **p < 0.01

The first simulated dataset simulated collagen fibril diameters measured from two different underlying unimodal distributions (Fig 1). The average fibril diameter was lower in group B compared to group A (*p* = 0.002; Fig 1B). In most instances, binned relative frequencies detected differences between groups although the p-values, location of the differences, and number of differences depended on bin width and location (Fig 1C). Notably, no differences were found between groups using a bin width of 45 nm and bin centers starting at 22.5 nm (*p* ≥ 0.054). Kernel density estimation was used to generate probability density functions for each sample, which were averaged across groups (Fig 1D). Comparison of these probability density functions using SnPM detected differences between groups, with three distinct suprathreshold clusters, which are shown as continuous shaded areas where the test statistic exceeds the critical test statistic (Fig 1E). There were more fibrils with diameters between 39.2 and 48.0 nm (*p* = 0.013) and between 59.3 and 74.6 nm (*p* = 0.004) in group A compared to group B, while there were fewer fibrils with diameters between 118.6 and 137.2 nm (p = 0.002) in group A compared to group B.

**Fig. 2:**
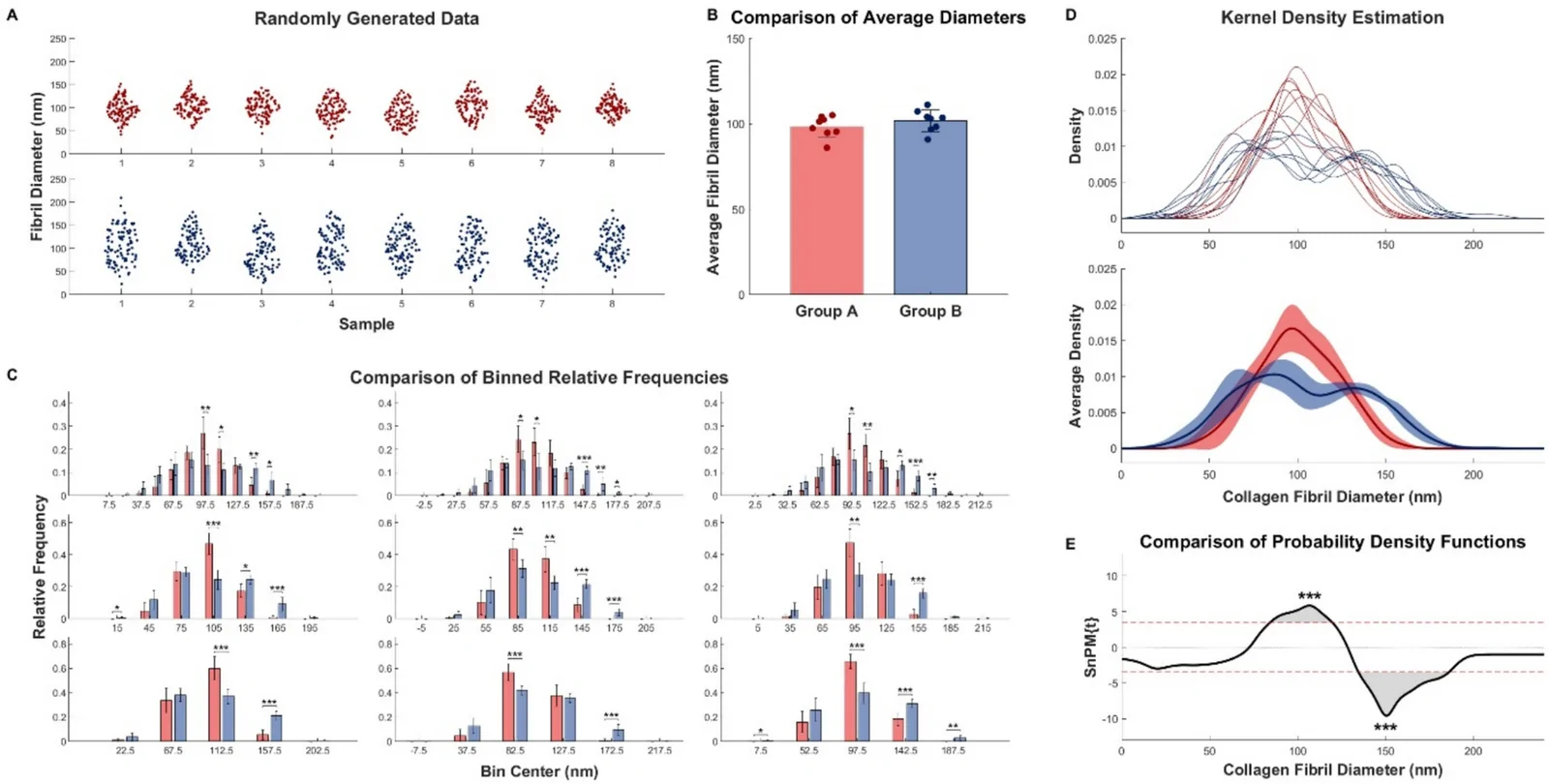
**A** Randomly generated bimodal data to simulate fibril diameter measurements. Each data point represents the diameter of a single collagen fibril, with 100 points per sample and 8 samples per group. **B** Comparison of average diameters did not detect a difference between groups. **C** Analysis of binned relative frequencies detected differences between groups but specific results depended on bin width and location. **D** Kernel density estimation generated sample-specific probability density functions. **E** Comparative analysis of probability density functions using SnPM detected and located differences between groups, where suprathreshold clusters are shown as continuous gray shaded areas that exceed the critical test statistic, shown as dashed red lines. Summary data shown as mean ± standard deviation. *p < 0.05, **p < 0.01, ***p < 0.001

### SnPM Detects Differences in Two Groups of Randomly Generated Bimodal Distributions

The second simulated dataset simulated collagen fibril diameters measured from two different underlying bimodal distributions (Fig 2). In this dataset, no difference was detected in the average fibril diameter between groups (*p* = 0.282; Fig 2B). Comparison of binned relative frequencies between groups found differences between groups, although again the *p*-values, location of differences, and number of differences were dependent on bin width and bin location (Fig 2C). Analysis using SnPM detected differences between groups with two suprathreshold clusters (Fig 2E). There were fewer fibrils with diameters between 84.2 and 119.8 nm (*p* < 0.001) and more fibrils with diameters between 134.5 and 186.1 nm (*p* < 0.001) in group A compared to group B.

### SnPM Detects Regional Differences in Two Groups of Fibril Diameter Distributions Within Mouse Supraspinatus Tendon

**Fig. 3:**
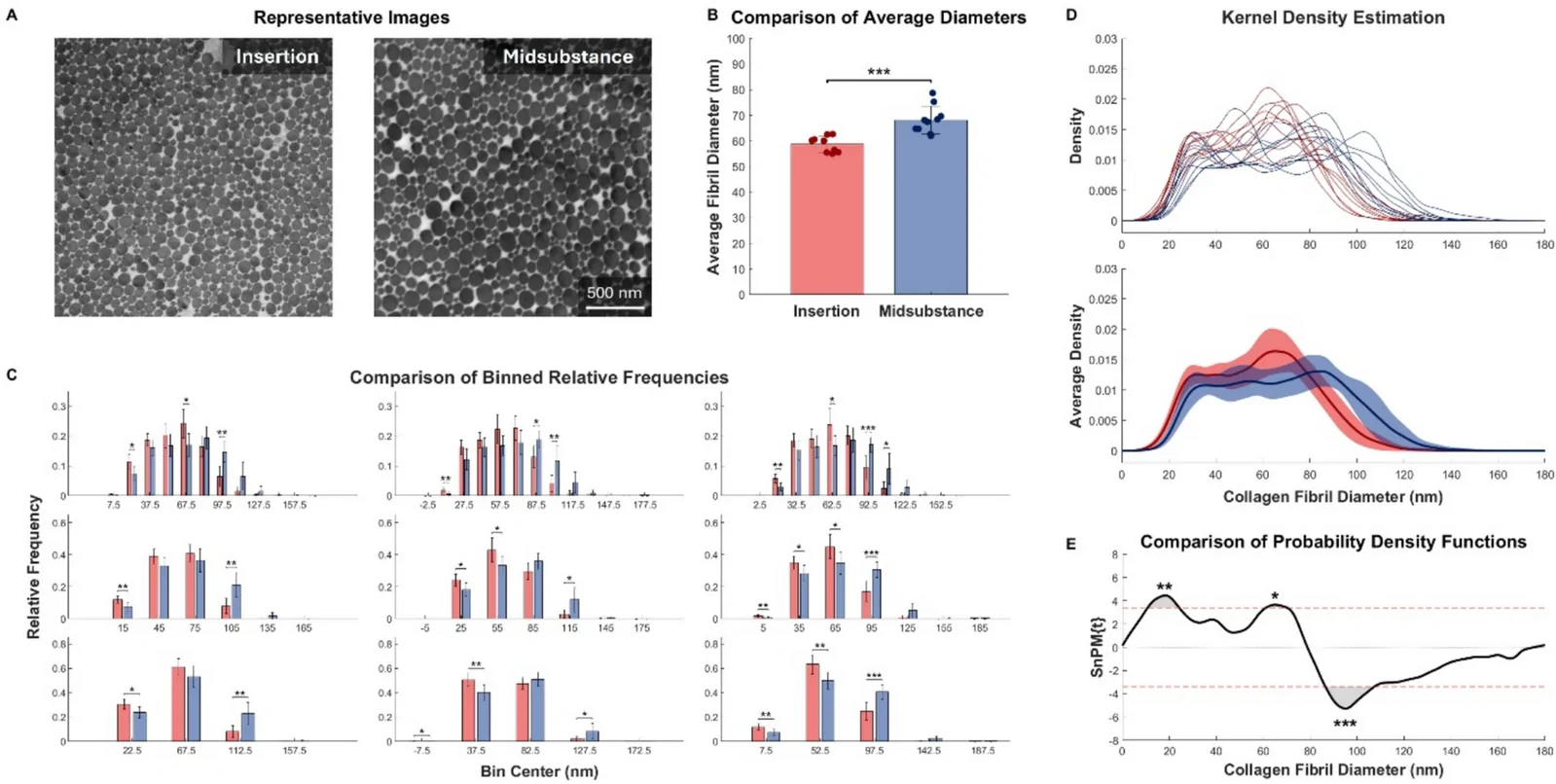
**A** Representative transmission electron microscopy images of supraspinatus tendon cross sections showing collagen fibrils within the insertion and midsubstance regions. **B** Comparison of average diameters detected a difference between regions. **C** Analysis of binned relative frequencies detected differences between regions but specific results depended on bin width and location. **D** Kernel density estimation generated sample-specific probability density functions. **E** Comparative analysis of probability density functions using SnPM detected and located differences between regions, where suprathreshold clusters are shown as continuous gray shaded areas that exceed the critical test statistic, shown as dashed red lines. Summary data shown as mean ± standard deviation. *p < 0.05, **p < 0.01, ***p < 0.001

Previously published experimental data measuring collagen fibril diameters in the insertion and midsubstance regions of mouse supraspinatus tendon were analyzed using the different statistical techniques. (Fig 3). The average fibril diameter was greater in the midsubstance compared to the insertion (*p* < 0.001; Fig 3B). Analysis using binned relative frequencies detected regional differences, although the statistical significance and location of the differences depended on bin width and bin location (Fig 3C). Using SnPM, the distributions were found to be different between the insertion and midsubstance regions with three suprathreshold clusters detected (Fig 3E). In the insertion, there were more fibrils with diameters between 10.7 and 24.3 nm (*p* = 0.003) and between 61.1 and 70.3 nm (*p* = 0.014), while the midsubstance had more fibrils with diameters between 86.5 and 107.3 nm (*p* < 0.001).

### SnPM Detects Age-Related Differences in Three Groups of Fibril Diameter Distributions Within Mouse Patellar Tendon

**Fig. 4:**
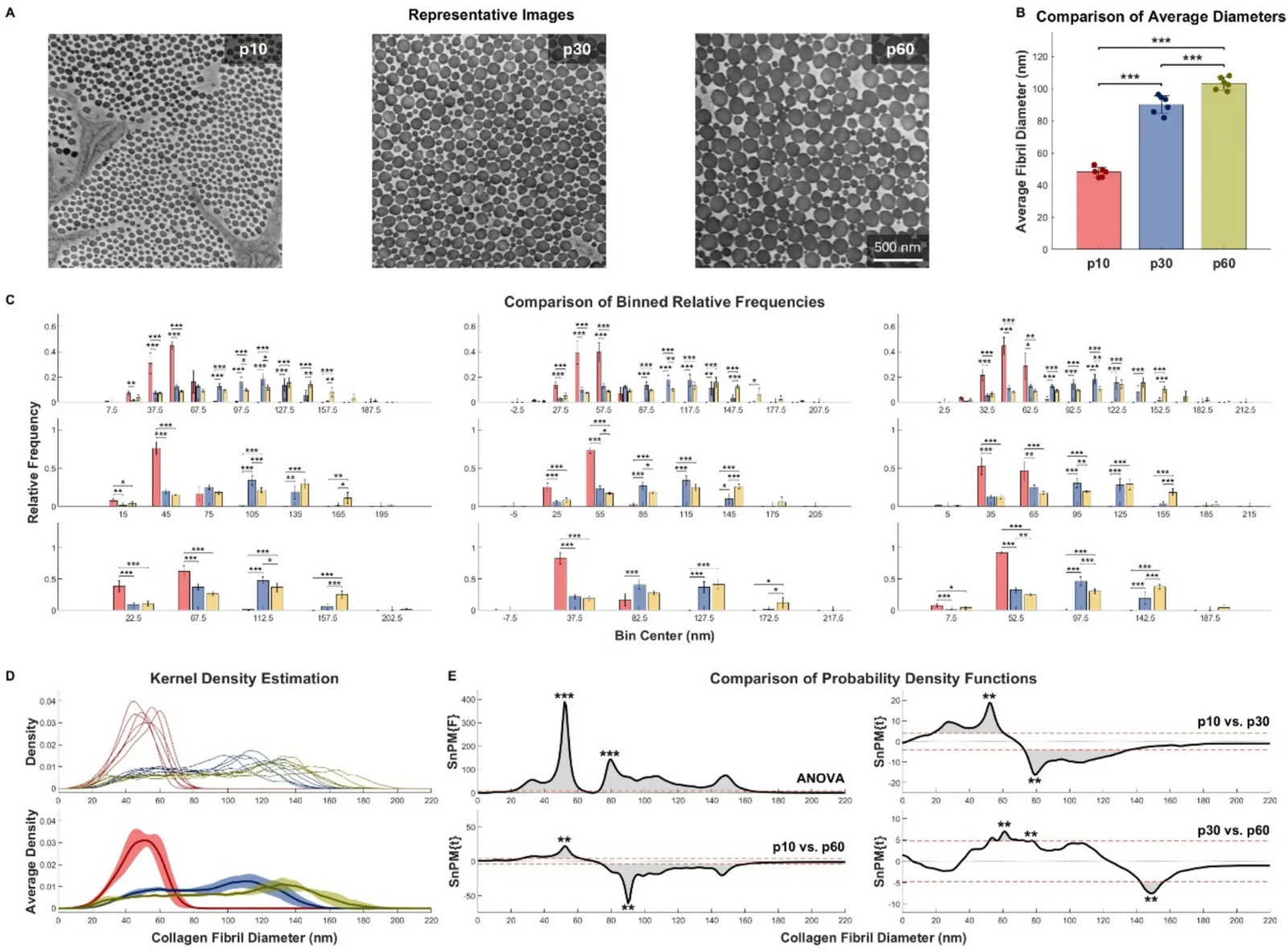
**A** Representative transmission electron microscopy images of patellar tendon cross sections showing collagen fibrils at postnatal days 10, 30, and 60. **B** Comparison of average diameters detected differences between ages. **C** Analysis of binned relative frequencies detected differences between ages but specific results depended on bin width and location. **D** Kernel density estimation generated sample-specific probability density functions. **E** Comparative analysis of probability density functions using SnPM detected and located differences between ages, where suprathreshold clusters are shown as continuous gray shaded areas that exceed the critical test statistic, shown as dashed red lines. Summary data shown as mean ± standard deviation. *p < 0.05, **p < 0.01, ***p < 0.001

A second previously published dataset measuring collagen fibril diameters in the patellar tendon from 10, 30, and 60-day-old mice were similarly analyzed (Fig 4). The average fibril diameter increased with increasing age (*p* < 0.001; Fig 4B). Age-related differences were also detected between binned relative frequencies of each group, although the significance and location of the differences again depended on bin width and bin location (Fig 4C). When analyzed using SnPM, the one-dimensional ANOVA found differences between groups (*p* < 0.001) that were probed further using post hoc analysis (Fig 4E). There were more fibrils with diameters between 18.0 and 60.7 nm (*p* = 0.002) and fewer fibrils with diameters between 72.8 and 135.9 nm (*p* = 0.001) in patellar tendons from 10-day-old compared to 30-day-old mice. Tendons from 10-day-old mice also had more fibrils with diameters between 25.8 and 62.2 nm (*p* = 0.002) and fewer fibrils with diameters between 74.2 and 160.2 nm (*p* = 0.001) compared to tendons from 60-day-old mice. Lastly, there were more fibrils with diameters between 51.8 and 73.5 nm (*p* = 0.002) and between 76.3 and 78.1 nm (*p* = 0.009) and fewer fibrils with diameters between 142.2 and 156.3 nm (*p* = 0.001) in tendons from 30-day-old mice compared to 60-day-old mice.

### Kernel Bandwidth Selection Method has Minimal Effect on Results from SnPM

The sensitivity of SnPM analysis of fibril diameter distributions to the kernel bandwidth selection method was determined by repeating the analyses presented above using the plug-in bandwidth selection method (Table 2, Figs. S1–S4). Qualitatively, the normal approximation method produced superior probability density functions that preserved the shape of the data, while smoothing out minor fluctuations that could still be seen when using the plug-in method in the experimental datasets due to the difference in kernel bandwidth. Still, the variation in kernel bandwidth from the two different methods had minimal effect on the results from SnPM analysis. The boundaries of suprathreshold clusters varied by a maximum of 3 nm and *p*-values changed by a maximum of 0.001 when similar clusters were detected. In one instance, in the comparison of distributions between p30 and p60 in patellar tendons, a suprathreshold cluster that only marginally exceeded the critical test statistic was broken up into multiple suprathreshold clusters over a similar range when using the plug-in kernel bandwidth selection method, which changes the appearance of the analysis but does little to affect the overall interpretation.

**Table 2:** Effect of kernel bandwidth selection methods on kernel bandwidth and suprathreshold clusters

	Comparison	Normal approximation method		Plug-in method	
		Kernel Bandwidth	Suprathreshold cluster range (*p*-value)	Kernel bandwidth	Suprathreshold cluster range (*p*-value)
Unimodal simulated	Group A vs. Group B	6.57	39.2–48.0 (0.013) 59.3–74.6 (0.004) 118.6–137.2 (0.002)	6.86	38.7–48.7 (0.012) 58.1–74.5 (0.004) 118.5–137.8 (0.002)
Bimodal simulated	Group A vs. Group B	7.67	84.2–119.8 (< 0.001) 134.5–186.1 (< 0.001)	7.05	85.2–119.5 (0.001) 134.5–185.1 (< 0.001)
Supraspinatus tendon experimental	Insertion vs. midsubstance	2.66	10.7–24.3 (0.003) 61.1–70.3 (0.014) 86.5–107.3 (< 0.001)	1.49	11.8–24.1 (0.004) 62.4–67.3 (0.015) 86.8–106.4 (< 0.001)
Patellar tendon experimental	p10 vs. p30	2.20	18.0–60.7 (0.002) 72.8–135.9 (0.001)	1.34	18.4–60.5 (0.002) 72.7–135.6 (0.001)
	p10 vs. p60		25.8–62.2 (0.002) 74.2–160.2 (0.001)		26.4–61.8 (0.002) 74.1–159.7 (0.001)
	p30 vs. p60		51.8–73.5 (0.002) 76.3–78.1 (0.009) 142.2–156.3 (0.001)		52.0–55.3 (0.004) 58.2–64.2 (0.002) 66.7–68.3 (0.009) 77.5–79.3 (0.009) 142.4–155.7 (0.001)

## Discussion

This study evaluated a novel application of SnPM in comparing differences in groups of collagen fibril diameter distributions. These data are essential for quantifying matrix assembly and remodeling processes, yet statistical analysis of these data is often hindered by limitations of conventional methods. One-dimensional analysis of probability density functions using SnPM overcame these limitations and detected differences in collagen fibril diameter distributions between groups, also locating those differences to specific ranges of fibril diameters without relying on any *a priori* decisions or input.

Among the alternative options to SnPM, the simplest is to extract a single value from the observed data, such as the average value, which can then be compared between groups using 0-dimensional statistical tests [17, 18]. In some cases, this can sufficiently capture changes in a distribution, such as a simple shift in a unimodal distribution, as demonstrated with our first simulated dataset (Fig. 1B). However, in more complex cases, a single metric will not be adequate. An average value does not accurately represent an asymmetric or multimodal distribution, and changes in such a distribution may not be reflected by a change in the average diameter, as demonstrated with our second simulated dataset (Fig. 2B). Even in situations where the average fibril diameter may be different between groups, this single metric does not capture the underlying changes in the distributions that led to this difference. Descriptive statistics other than the average value, such as the standard deviation, could also be used, but are subject to the same limitations. This option remains valuable for studies that have a hypothesis that can be tested using a single value and that will not be negatively impacted by missing changes that occur unrelated to that hypothesis; for instance, a study where it is hypothesized that a treatment will increase the average fibril diameter, and any other potential changes, such as a change in the range of fibril diameters, would not be scientifically meaningful. However, most studies will benefit from a broader statistical test that does not require such specific hypotheses and can compare the full distributions of collagen fibril diameters.

Analysis of binned relative frequencies is another option that has been used in previous studies [18–20]. This approach resembles a comparison of the full distributions, but still reduces the data into scalar values that can be compared using 0-dimensional tests. In addition to the limitations that are inherent in such data reduction [21], results from this analysis are dependent on bin width and location choice, as shown in all of our datasets. When there is scientific justification for the bins used, this could be a valid approach. In most studies, however, the choices for binning parameters are arbitrary, which can lead to researcher-dependent results and increase the risk of p-hacking in borderline cases where the choice of binning parameters might be the deciding factor between a positive and negative result [22].

Other analysis methods not shown in this paper have also been used to compare fibril diameters. For instance, the Kolmogorov–Smirnov test which has also been adopted by some for comparing fibril diameter distributions is specifically used to compare differences in probability distributions [23–25]. However, this test compares only two distributions, not groups of distributions, and therefore, to use this test, the data must be pooled across biological samples to create a single distribution for each experimental group. This data pooling eliminates the biological variability between samples that is important for comparison between groups, potentially leading to false-positive results when the variability within groups is comparable or greater than the variability between groups. Analysis using other statistical tests with similar data pooling across samples similarly eliminates this important variability between biological samples [26, 27].

Another approach involves fitting a given distribution function to the observed data, such as a set of Gaussian curves [28, 29]. The mean and variance for each Gaussian can then be determined and compared between experimental groups. However, rigorous use of this approach requires choosing a distribution to fit to the data *a priori*, which may or may not provide a good fit to the experimental data. In addition, comparison of these parameters requires the overall shape of the distributions to be the same between all experimental groups and between all samples within each group. These strict requirements render this approach nonideal for most experimental data where the shape of the distribution can vary between samples or between experimental groups.

Analysis of collagen fibril diameters using SnPM overcomes the limitations of these alternative methods. It is a true one-dimensional analysis, allowing for the evaluation of differences in the full distributions of fibril diameters through the use of kernel density estimation. Consequently, it does not rely on specific hypotheses regarding individual metrics that can be extracted from the full set of observations. In contrast to the comparison of binned relative frequencies, it is independent of arbitrary choices regarding bin width or location, and it is insensitive to changes in kernel bandwidth from alternate bandwidth selection methods. Furthermore, it properly accounts for biological variability between samples and does not require any *a priori* assumptions about the shape of the distributions. Therefore, analysis using SnPM instead provides the exact ranges of fibril diameters where the probability density is different between groups without the need for additional input. However, some potential limitations should also be noted. Estimating probability density functions for each sample requires sufficient observations to be confident in the kernel density estimation. With automated analysis of images that can contain hundreds of fibrils each, this condition is met relatively easily [30, 31]. In addition, SnPM used in this way compares the values of probability density functions at different diameters but does not directly answer questions about changes in specific metrics, such as the average diameter. In cases where these questions are of interest, additional statistical tests could be conducted alongside of, or instead of SnPM to address more specific hypotheses. As with any other statistical test, SnPM also does not provide any information regarding the scientific meaning of significant differences, which must be ascertained by the researcher.

In this study, we demonstrated the use of SnPM to compare two or three groups of probability density functions using the t-statistic or F-statistic. Still, applications for SnPM go beyond what was demonstrated. The open-source package spm1d is equipped with an array of 1-dimensional statistical tests for more complex study designs with more study groups or multiple independent variables. Alternative choices for the test statistic could be used, each of which would need to have its strengths and weaknesses, including its effect on statistical power, considered for the particular application [32]. In addition, while this study focused on measurements of fibril diameter, the applications for SnPM in biomedical engineering research are much broader. Even with respect to collagen fibrils, additional parameters such as circularity or solidity could be measured and analyzed using the same method. More generally, SnPM could be used in this manner for any dataset where there are sufficient distinct observations per sample to estimate a probability density function for each biological sample, and it would be of scientific interest to compare probability density functions between groups. Other potential applications of SnPM or SPM extend even farther to any 1-dimensional dataset where a measured property varies based on continuous independent variable, such as measuring alignment of collagen as a function of mechanical strain or measuring changes in cell shape over time [33, 34]. Analysis using SnPM, therefore, has valuable utility in comparing collagen fibril diameter distributions and promising potential for numerous other applications within biomedical engineering research.

## Supplementary Information

Below is the link to the electronic supplementary material.Supplementary file1 (PDF 879 kb)

## Data Availability

The raw data and MATLAB scripts used for the preparation of this manuscript are available on GitHub at https://github.com/jeremyeekhoff/Statistical-nonParametric-Mapping-of-Collagen-Fibril-Diameter-Distributions.
