# Supplementary material for: Statistical nonParametric Mapping Enables Rigorous Comparison of Collagen Fibril Diameter Distributions
Source: Ann Biomed Eng. 2026 Mar 4;54(9):2937–46. doi: 10.1007/s10439-026-04064-2 (PMC13451988; doi:10.1007/s10439-026-04064-2)
Supplement: Supplementary file 1 [file 10439_2026_4064_MOESM1_ESM.pdf]

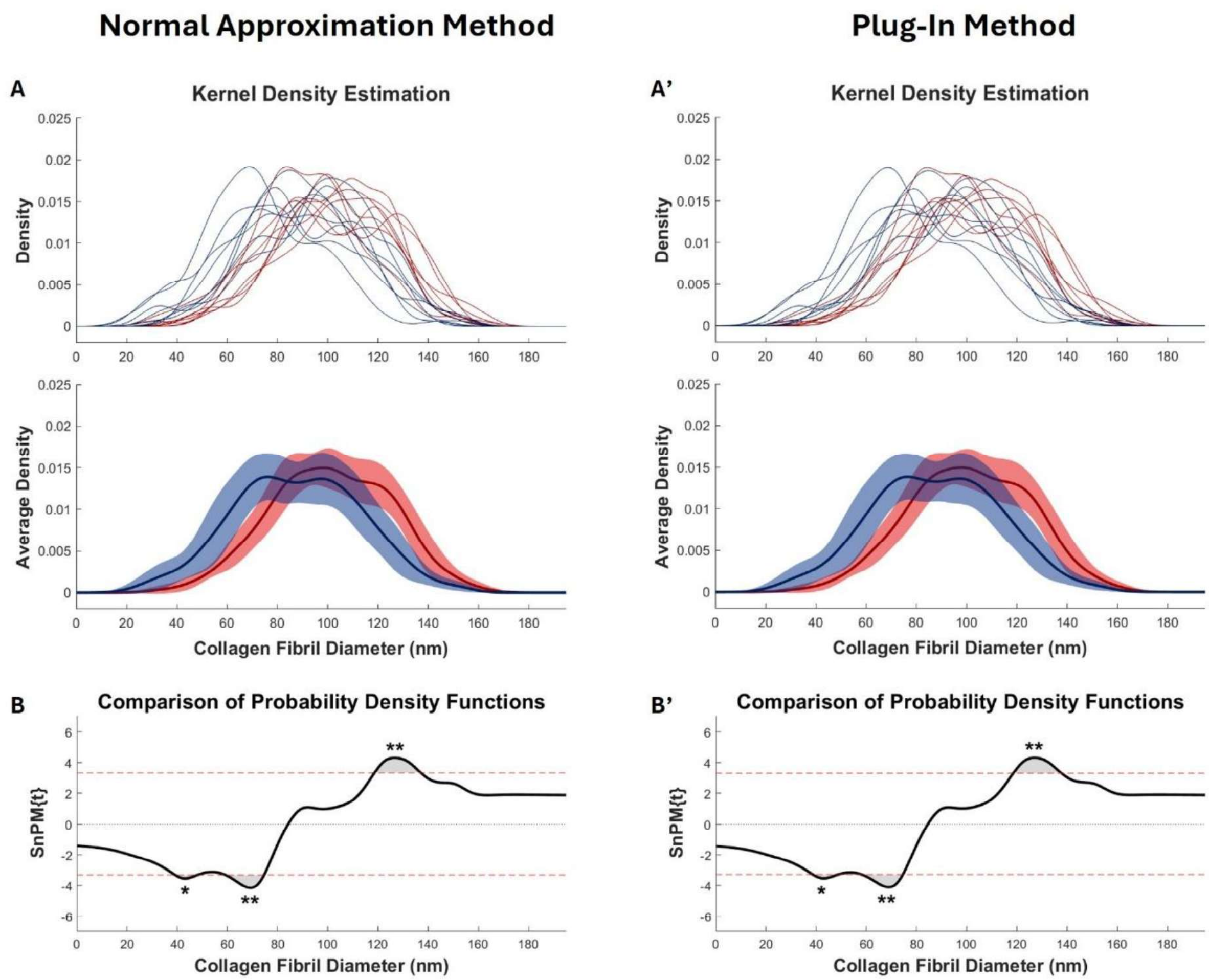

Figure S1. Kernel density estimation of the unimodal simulated dataset using the normal approximation (A) and plug-in (A') bandwidth selection methods. (B, B') SnPM analysis of the corresponding probability density functions.

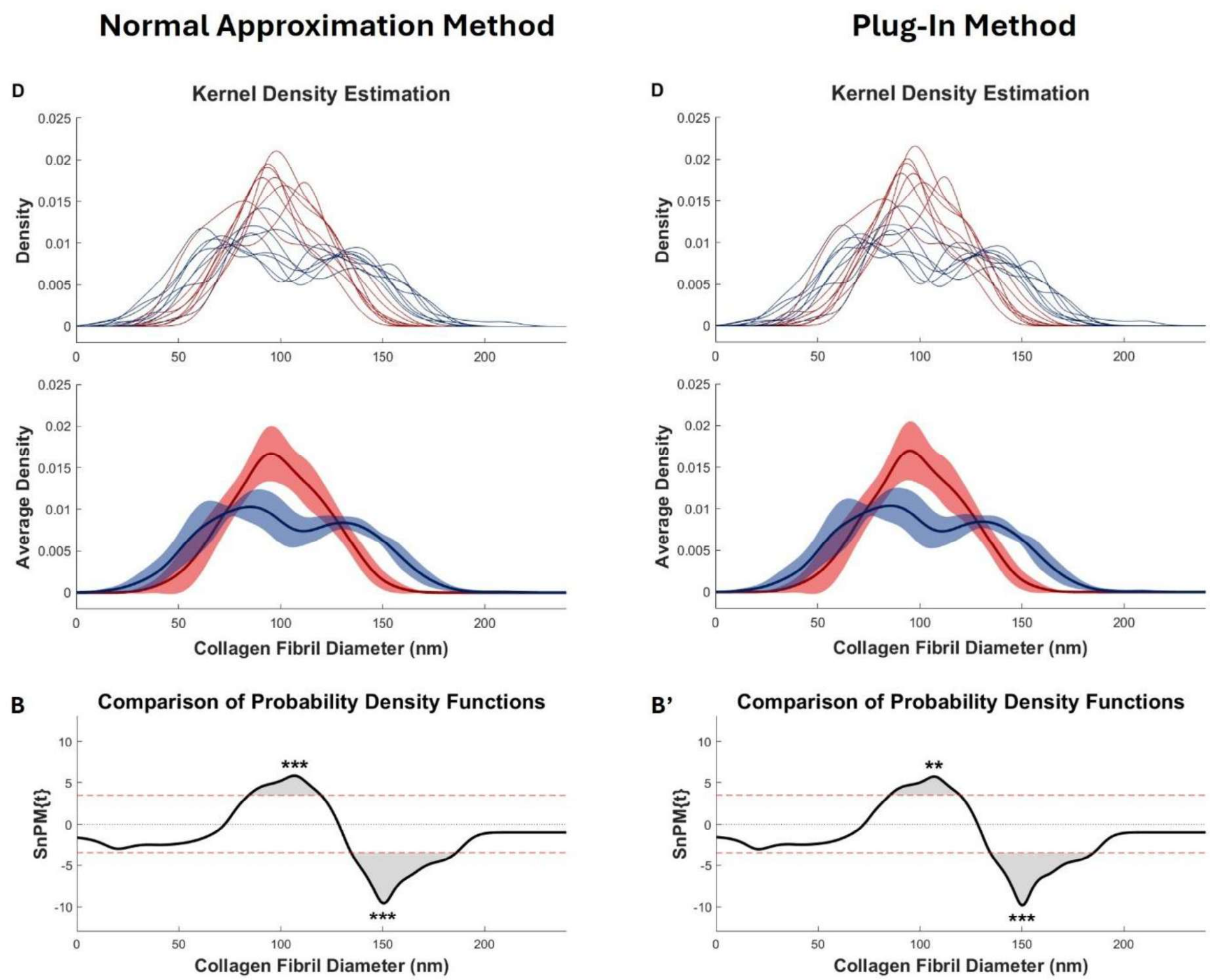

Figure S2. Kernel density estimation of the bimodal simulated dataset using the normal approximation (A) and plug-in (A') bandwidth selection methods. (B, B') SnPM analysis of the corresponding probability density functions.

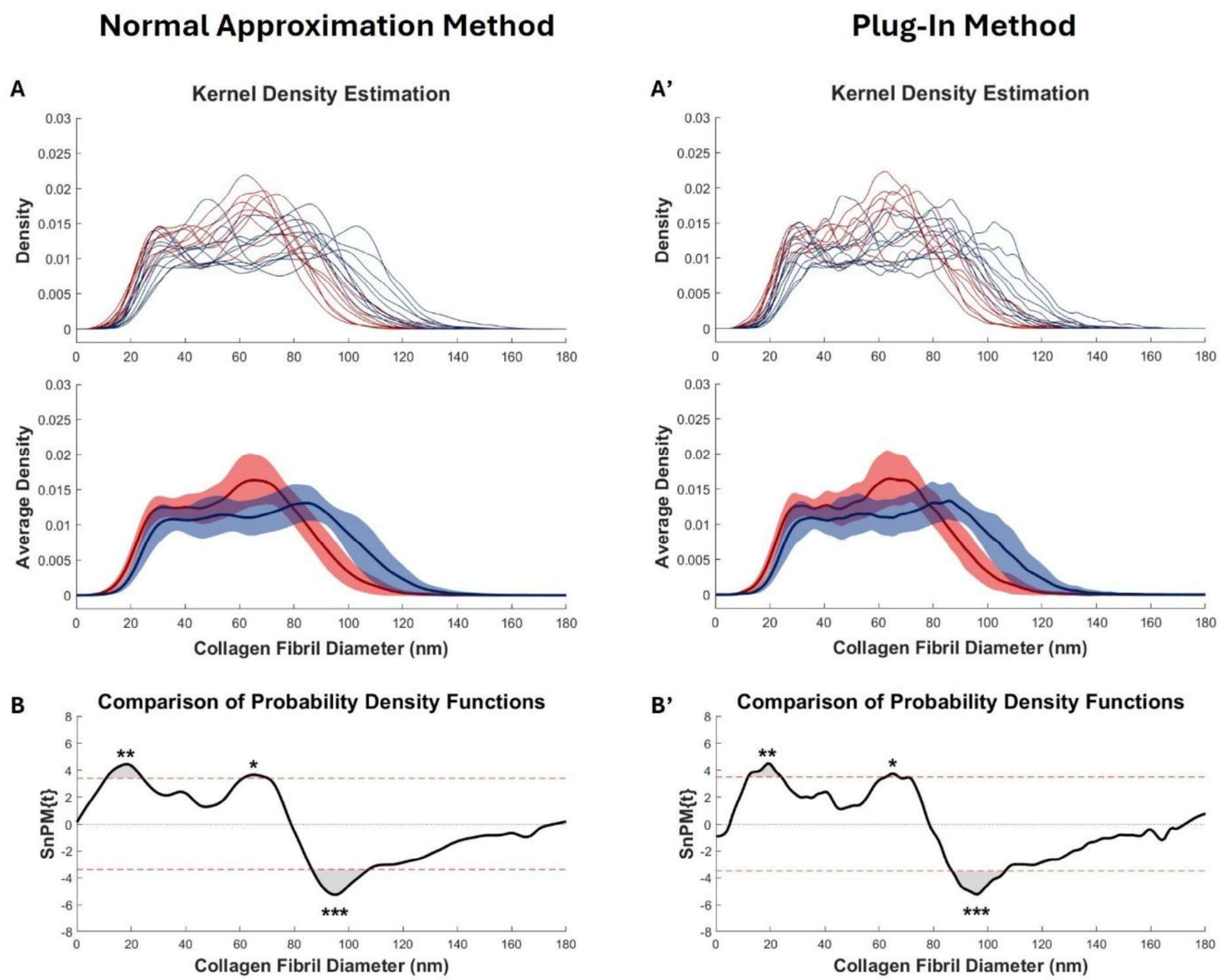

Figure S3. Kernel density estimation of the supraspinatus tendon experimental dataset using the normal approximation (A) and plug-in (A’) bandwidth selection methods. (B, B’) SnPM analysis of the corresponding probability density functions.

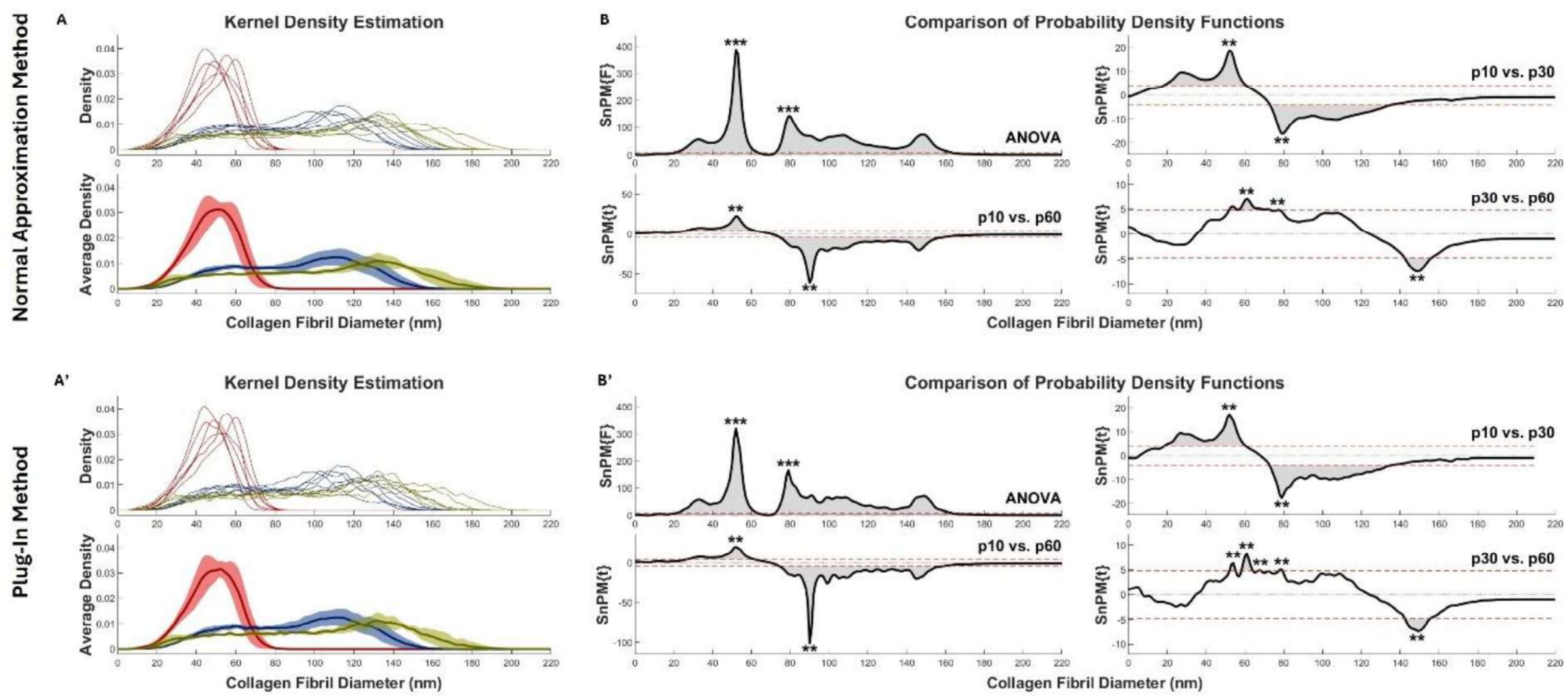

Figure S4. Kernel density estimation of the patellar tendon experimental dataset using the normal approximation (A) and plug-in (A') bandwidth selection methods. (B, B') SnPM analysis of the corresponding probability density functions.
